# Chemokine receptor CXCR4 oligomerization is disrupted selectively by the antagonist ligand IT1t

**DOI:** 10.1074/jbc.RA120.016612

**Published:** 2020-12-06

**Authors:** Richard J. Ward, John D. Pediani, Sara Marsango, Richard Jolly, Michael R. Stoneman, Gabriel Biener, Tracy M. Handel, Valerică Raicu, Graeme Milligan

**Affiliations:** 1 Centre for Translational Pharmacology, Institute of Molecular, Cell and Systems Biology, College of Medical, Veterinary and Life Sciences, University of Glasgow, Glasgow, Scotland, United Kingdom; 2 Physics Department, University of Wisconsin-Milwaukee, Milwaukee, Wisconsin, USA; 3 Skaggs School of Pharmacy and Pharmaceutical Sciences, University of California, San Diego, La Jolla, California, USA

**Keywords:** chemokine, confocal microscopy, dimerization, G protein–coupled receptor (GPCR), microscopic imaging, oligomerization, tertiary structure, BRET, bioluminescence resonance energy transfer, FIF, fluorescence intensity fluctuation, GPCRs, G protein–coupled receptors, IT1t, isothiourea-1t, mEGFP, monomeric enhanced green fluorescent protein, MEU, monomeric equivalent unit, PSF, point spread function, QB, quantal brightness, RoIs, regions of interest, SD, standard deviation, SpIDA, spatial intensity distribution analysis

## Abstract

CXCR4, a member of the family of chemokine-activated G protein–coupled receptors, is widely expressed in immune response cells. It is involved in both cancer development and progression as well as viral infection, notably by HIV-1. A variety of methods, including structural information, have suggested that the receptor may exist as a dimer or an oligomer. However, the mechanistic details surrounding receptor oligomerization and its potential dynamic regulation remain unclear. Using both biochemical and biophysical means, we confirm that CXCR4 can exist as a mixture of monomers, dimers, and higher-order oligomers in cell membranes and show that oligomeric structure becomes more complex as receptor expression levels increase. Mutations of CXCR4 residues located at a putative dimerization interface result in monomerization of the receptor. Additionally, binding of the CXCR4 antagonist IT1t—a small drug-like isothiourea derivative—rapidly destabilizes the oligomeric structure, whereas AMD3100, another well-characterized CXCR4 antagonist, does not. Although a mutation that regulates constitutive activity of CXCR4 also results in monomerization of the receptor, binding of IT1t to this variant promotes receptor dimerization. These results provide novel insights into the basal organization of CXCR4 and how antagonist ligands of different chemotypes differentially regulate its oligomerization state.

In recent times, structural studies have provided atomic-level details of the organization of the 7-transmembrane domain architecture of many members of the rhodopsin-like class A family of G protein–coupled receptors (GPCRs), as well as the basis of binding of various ligands to them and their interactions with signaling proteins (1, 2). However, features of GPCRs that have remained largely recalcitrant to such approaches are the extent and nature of their quaternary structures, and whether and how quaternary structure is regulated by ligand binding, both of which remain highly contentious topics (3, 4, 5). It has become evident, however, that within the membrane environment, many potential GPCR quaternary complexes are transient or metastable (6), existing over timescales of seconds. It is thus possible that the binding of ligands might modulate receptor–receptor interactions. The process of extracting GPCRs from cell membranes and purifying them with detergent for crystallography is not conducive to maintaining noncovalently linked protein–protein interactions. As such, although apparent “dimers” of class A GPCRs have been observed in a significant number of X-ray–based structures (7, 8, 9), the physiological relevance of these structures is uncertain. Indeed, Robertson *et al*. (9) highlighted specifically the challenges of deconvoluting physiologically relevant “dimerization” interfaces from those that simply mediate crystal contacts, and this question has recently been the subject of review (10).

Chemokine receptors represent a family of GPCRs for which there is strong evidence of dimerization (11). CXCR4 has been particularly well studied because of its roles in cancer and viral infections, including its function as a coreceptor for strains of the HIV-1 virus (12, 13, 14). Moreover, blockers of the receptor are used clinically, whilst others are in development, making it a particularly compelling receptor to address how the extent and degree of oligomeric organization of a GPCR may be correlated with expression levels and regulated by ligand binding. Several atomic-level structures of CXCR4 reveal the dimeric organization of the receptor with a clearly defined interface between the monomers (7). Furthermore, in early studies using bioluminescence resonance energy transfer (BRET), Babcock *et al*. (15) demonstrated receptor dimerization/oligomerization that was reported to be unaffected by CXCR4 expression level or the presence of ligands, whereas Percherancier *et al*. (16) indicated that ligand binding might alter the conformation of CXCR4 receptor complexes without directly disrupting dimeric structure. Subsequently, this same group expanded their studies to indicate that higher-order oligomers of the CXCR4 receptor can also exist (17), and similar conclusions have been reached by others using distinct approaches (18). By contrast, in single-molecule imaging studies, Lao *et al*. (19) suggested the basal state of the receptor to be largely monomeric at low expression levels but that a fraction of the receptor was either dimeric or oligomeric at increased receptor levels.

Both approaches mentioned previously have inherent limitations. In the single-molecule imaging studies, expression levels were extremely low (stated to be <2 molecules.μm^−2^; (19)), and once expression level increased significantly, single-molecule imaging became problematic. By contrast, in the BRET-based studies, levels of expression are often poorly defined, and it is challenging to quantify the extent of dimerization and to distinguish dimers from oligomers.

As an alternative approach, the analysis of fluorescence fluctuations has begun to be recently explored to quantify protein dimers and oligomers and their regulation (20). Such approaches have advantages over resonance energy transfer studies including BRET because only a single fluorophore-modified species needs to be expressed. Compared with single-molecule imaging studies, they also allow analysis over a substantially wider expression range and at higher expression levels. One method that analyses fluorescence fluctuations and that has been applied to assess GPCR quaternary organization in cells is spatial intensity distribution analysis (SpIDA) (21). Herein, we initially use SpIDA to assess the oligomeric organization of CXCR4 across a defined range of expression levels and how it is affected by the binding of CXCR4 antagonists. However, because SpIDA, and other methods that assess fluorescence fluctuations, has specific limitations in that they only provide average oligomer sizes from interrogated regions of interest (RoIs), which may contain complex mixtures of oligomers of varying size, we have also applied a recently developed technique, fluorescence intensity fluctuation (FIF) spectrometry, which is able to overcome this and other challenges (22, 23). In parallel, we provide a biochemical measure of receptor oligomerization by employing nondenaturing blue native-PAGE to measure ligand-induced effects on CXCR4 organization. Overall, these studies show that CXCR4 is significantly organized as a dimer, and that oligomeric organization increases further with increasing receptor expression. Whilst the CXCR4 small molecule antagonist isothiourea-1t (IT1t) rapidly and reversibly promotes monomerization of the receptor, the clinically employed CXCR4 antagonist AMD3100 (24) was not effective in this regard. We also show that specific mutations altering a predicted dimerization interface in transmembrane domain V identified in the atomic-level X-ray structures of Wu *et al*. (7), are indeed able to limit or almost fully abrogate CXCR4 dimerization, whilst dimerization of a constitutively active mutant of CXCR4 is instead actively promoted by binding of IT1t. Because certain mutants of CXCR4 that are largely if not completely monomeric are still able to bind the native chemokine agonist CXCL12, and thence activate G_i_-family G proteins, with potency similar to the wildtype receptor, we conclude that both monomers and dimers of the CXCR4 receptor are competent to transduce G protein–mediated signaling.

## Results

### Expression and quantification of CXCR4–mEGFP

**Figure 1:**
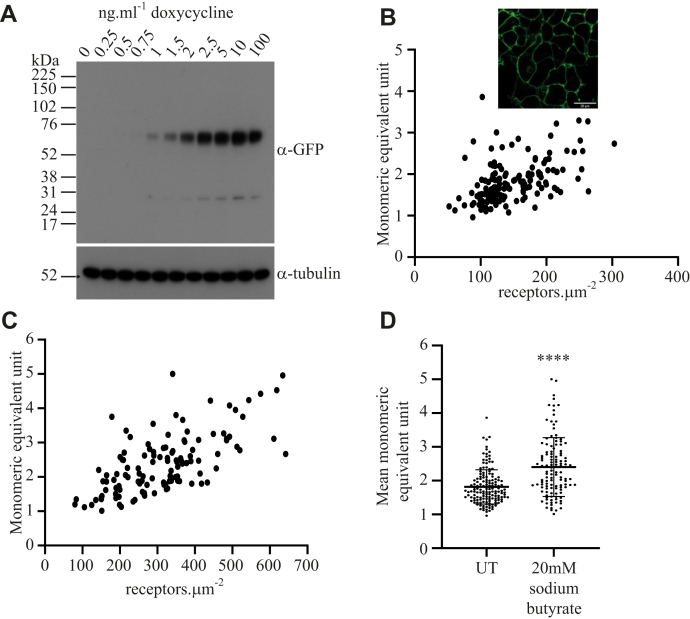
**Oligomeric organization of CXCR4–monomeric enhanced green fluorescent protein (mEGFP) determined by spatial intensity distribution analysis.** A Flp-In T-REx 293 doxycycline-inducible cell line was generated to express human CXCR4–mEGFP. *A, upper panel*, representative anti-GFP immunoblot of lysates from cells induced with the indicated concentrations of doxycycline for 24 h; *lower panel*, antitubulin immunoblot of the same samples. *B (inset)*, confocal image of cells induced with 100 ng.ml^−1^ doxycycline. The scale bar represents 20 μm. *B,* spatial intensity distribution analysis performed on the CXCR4–mEGFP expressing cell line induced by treatment with 100 ng.ml^−1^ doxycycline. *C,* spatial intensity distribution analysis of the CXCR4–mEGFP cell line induced by 100 ng.ml^−1^ doxycycline and treated overnight with 20 mM sodium butyrate (for quantitative details of *B* and *C*, see Table 1). *D,* monomeric equivalent units with and without treatment with 20 mM sodium butyrate (untreated [UT] = 1.82 ± 0.51, n = 150; 20 mM sodium butyrate = 2.40 ± 0.87, n = 120; means ± SD, ∗∗∗∗*p* < 0.0001).

To explore the organizational structure of the CXCR4 receptor in a cellular environment and how this might vary with expression level, we stably expressed a CXCR4–monomeric enhanced green fluorescent protein (CXCR4–mEGFP) fusion construct in Flp-In T-REx 293 cells. This allowed expression of the receptor construct to be controlled in a doxycycline-regulated manner. Addition of differing concentrations of doxycycline 24 h before cell harvest resulted in the expression of varying amounts of CXCR4–mEGFP as assessed by immunoblotting lysates of such cells with an anti-GFP antiserum following their resolution by SDS–PAGE (Fig. 1*A*). As anticipated, although undetectable without doxycycline treatment, following induction by doxycycline, CXCR4–mEGFP was expressed and migrated as an apparent single species with M_r_ close to 70 kDa (Fig. 1*A*). Low levels of an approximately 27 kDa immunoreactive species, which might reflect very limited proteolysis of the CXCR4–mEGFP construct, were also detected by the anti-GFP antiserum (Fig. 1*A*). Parallel immunoblotting to detect α-tubulin provided a suitable loading control (Fig. 1*A*). Confocal imaging of cells that had been induced to express CXCR4–mEGFP indicated that the construct was located predominantly at the cell surface (Fig. 1*B* inset). In recent times, various methods based on analysis of fluorescence fluctuations have been used to assess the oligomeric organization of a range of fluorophore-tagged proteins, including members of the GPCR superfamily. Using SpIDA (see Methods section [Equation 2] and Ref. (21)), we were able to quantify the number of CXCR4–mEGFP.μm^−^^2^ in defined RoIs within the basolateral surface of these cells after receptor induction with varying concentrations of doxycycline (Fig. 1*B*). The density range was substantial, from approximately 50 receptors·μm^−2^ to approximately 300 receptors·μm^−2^. By comparison with the measured quantal brightness (QB) of a monomeric control in which single copies of mEGFP were linked to the plasma membrane *via* a lipidated peptide sequence (PM-1–mEGFP) and also expressed stably in Flp-In T-REx 293 cells (25, 26), CXCR4–mEGFP was shown to exist predominantly as dimers/oligomers across this expression range (Fig. 1*B*). Furthermore, a clear positive correlation was observed over this range between expression level and oligomeric complexity (Fig. 1*B*). To expand the relationship between expression levels and oligomeric complexity, in a number of experiments, we treated cells with the histone deacetylase inhibitor sodium butyrate (20 mM) as well as with doxycycline because butyrate treatment is known to be able to upregulate levels of protein expressed from a cytomegalovirus promoter (27, 28). This indeed resulted in higher levels of expression of CXCR4–mEGFP as quantified by SpIDA in defined RoIs (Fig. 1*C* and Table 1). Such a treatment also resulted in greater mean oligomeric complexity (*p* < 0.0001) (Fig. 1*D*) and is consistent with the potential of CXCR4 at higher levels of expression to organize as oligomeric complexes that are greater than dimers.

**Table 1:** Summary of SpIDA data values

Construct	Treatment	MEU (mean ± SD)	Receptors.μm^−^^2^ (mean ± SD)	RoIs (n)	% Monomer	% Dimer/oligomer	Figures
CXCR4–mEGFP		1.82 ± 0.51	147.80 ± 47.73	150	12.00	88.00	1, 3
CXCR4–mEGFP	2 × 10^−2^ M sodium butyrate	2.40 ± 0.87	314.76 ± 124.88	120	5.83	94.17	1
CXCR4–mEGFP	2 × 10^−8^ M IT1t	1.17 ± 0.22	124.92 ± 40.30	150	77.33	22.67	3
CXCR4–mEGFP	1 × 10^−6^ M AMD3100	1.77 ± 0.59	121.98 ± 47.37	150	24.67	75.33	3
Trp^195^AlaCXCR4–mEGFP		1.24 ± 0.34	110.04 ± 37.12	100	70.00	30.00	5
Leu^194^Ala, Trp^195^Ala, Leu^267^Ala, Glu^268^AlaCXCR4–mEGFP		1.00 ± 0.21	62.90 ± 15.74	100	91.00	9.00	5
Asn^119^LysCXCR4–mEGFP		1.03 ± 0.22	160.63 ± 34.40	50	88.00	12.00	6
Asn^119^LysCXCR4–mEGFP	2 × 10^−8^ M IT1t	1.56 ± 0.34	219.63 ± 44.08	50	32.00	68.00	6

IT1t, isothiourea-1t; mEGFP, monomeric enhanced green fluorescent protein; MEU, monomeric equivalent unit; RoIs, regions of interest; SD, standard deviation; SpIDA, spatial intensity distribution analysis.

Each RoI was taken from an individual distinct cell, and the total number of RoIs is from (at least) three coverslips, each derived from independent passages for the relevant cell line. Monomeric equivalent unit is compared with the quantal brightness of PM1–mEGFP.

### IT1t disrupts CXCR4 dimers and oligomers

**Figure 2:**
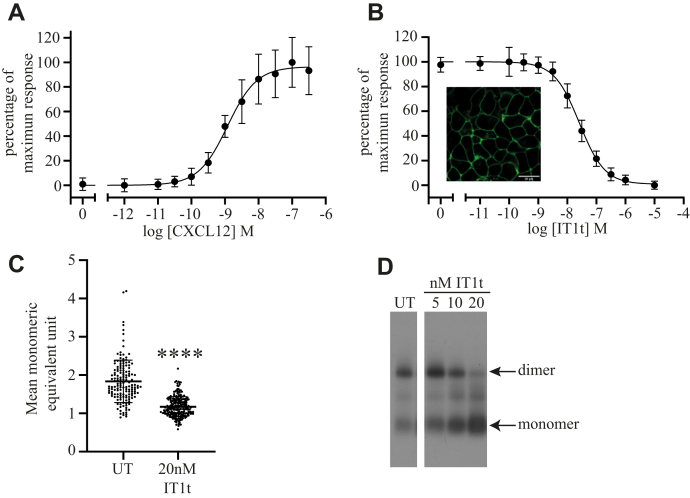
**The antagonist IT1t dihydrochloride disrupts CXCR4 oligomeric structure after long-term exposure**. *A,* membranes prepared from cells as in Figure 1 and induced with 100 ng.ml^−1^ doxycycline (24 h) were exposed to the agonist CXCL12 at the concentrations indicated. [^35^S]GTPγS binding is plotted as a percentage of maximum response (EC_50_ = 1.19 × 10^−9^ M). *B,* as *A*, but CXCL12 was added at EC_80_ concentration (8.11 × 10^−9^ M) along with varying concentrations of IT1t. Combined results of n = 4 experiments performed on separate membrane preparations. *B (inset),* confocal image of cells induced with 100 ng.ml^−1^ doxycycline (24 h) and treated with 20 nM IT1t for 3 h. The scale bar represents 20 μm. *C,* monomeric equivalent units, measured by spatial intensity distribution analysis, with and without treatment with 2 × 10^−8^ M IT1t (untreated [UT] = 1.82 ± 0.51, 2 × 10^−8^ M; IT1t = 1.17 ± 0.22, means ± SD; both n = 150; ∗∗∗∗*p* < 0.0001). *D,* blue native-PAGE of lysates of CXCR4–monomeric enhanced green fluorescent protein expressing cells (UT) or treated with the indicated concentrations of IT1t, positions of monomeric and dimeric bands as indicated. IT1t, isothiourea-1t.

**Figure 3:**
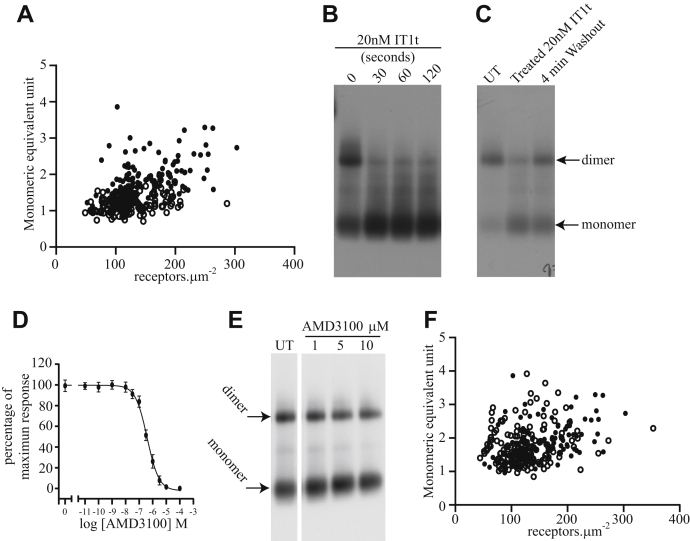
**Effects of IT1t on CXCR4–monomeric enhanced green fluorescent protein (mEGFP) dimerization are rapid and reversible in contrast to AMD3100, which does not promote monomerization**. *A,* spatial intensity distribution analysis of CXCR4–mEGFP cell line induced by 24 h addition of 100 ng.ml^−1^ doxycycline (*closed circles* untreated, data as described for Figure 2*A*; data collected and analyzed in parallel, *open circles* treated with 2 × 10^−8^ M IT1t for 2 min, for details, see Table 1). *B* and *C,* blue native-PAGE anti-GFP immunoblots of lysates of CXCR4–mEGFP expressing cells. *B,* untreated (0) or treated with 2 × 10^−8^ M IT1t for the times indicated. *C,* CXCR4–mEGFP expressing cells either untreated (UT) or treated with 2 × 10^−8^ M IT1t for 2 h (treated) and then removed by changing the medium and collected 4 min later (washout). Positions of monomer and dimer bands indicated. *D*, AMD3100 antagonizes CXCL12-induced binding of [^35^S]-GTPγS in membranes of CXCR4–mEGFP expressing cells in a concentration-dependent manner. Data are combined results of n = 3 experiments. *E,* anti-GFP immunoblots following blue native-PAGE of lysates of CXCR4–mEGFP expressing (100 ng.ml^−1^ dox) cells treated with the indicated concentrations of AMD3100 for 1 h: UT = untreated. Monomer and dimer bands indicated. *F*, spatial intensity distribution analysis of cells treated for 2 min with 1 × 10^−6^ M AMD3100, *open circles.* Parallel UT data from Figure 2*A* shown for comparison, *closed circles* (see also Table 1). IT1t, isothiourea-1t.

Whether ligands affect the oligomeric organization of CXCR4 remains uncertain. However, in at least one of the dimeric atomic-level structures of CXCR4 (Protein Data Bank 3ODU), an associated molecule of the antagonist ligand IT1t is shown bound to each of the interacting protomers (7). The best studied endogenous agonist of CXCR4 is the chemokine CXCL12 (previously designated SDF1α). This chemokine promoted binding of [^35^S]GTPγS in membranes of Flp-In T-REx 293 cells induced to express CXCR4–mEGFP in a concentration-dependent manner with an EC_50_ of 1.6 ± 0.2 × 10^−9^ M (mean ± SD; n = 4) (Fig. 2*A*). The affinity of IT1t (*K*_i_ = 5.2 ± 0.1 × 10^−9^ M) for the CXCR4–mEGFP construct was calculated (see Methods section) from the potency of IT1t to prevent binding of [^35^S]GTPγS induced by an EC_80_ concentration of CXCL12 (Fig. 2*B*). Sustained treatment (16 h) of cells induced to express CXCR4–mEGFP with 20 nM IT1t did not noticeably alter the distribution pattern of the receptor construct as this remained at the cell surface (Fig. 2*B* inset). However, analysis by SpIDA (Fig. 2*C*) indicated that the median organization of the receptor was substantially less oligomeric after such treatment (*p* < 0.0001) (Fig. 2*C* and Table 1). To complement this conclusion, we resolved membranes of Flp-In T-REx 293 cells induced to express CXCR4–mEGFP that had been either untreated or treated with varying concentrations of IT1t for 16 h using nondenaturing blue native-PAGE and then immunoblotted to detect mEGFP (Fig. 2*D*). Whilst 5 × 10^−9^ M IT1t had little effect on the proportion of CXCR4–mEGFP migrating as an apparent monomer or dimer, very marked differences in the migration pattern were observed after treatment with either 1 × 10^−8^ or 2 × 10^−8^ M IT1t. Whereas most of the construct from untreated cells or those treated with 5 × 10^−9^ M IT1t migrated consistent with CXCR4–mEGFP being dimeric, after treatment with either 1 × 10^−8^ M or 2 × 10^−8^ M IT1t, most of the receptor migrated more rapidly, consistent with it being a monomer, and this was more profound at 2 × 10^−8^ M IT1t (Fig. 2*D*). As the effective concentrations are 2 to 4 times the measured binding affinity of IT1t for CXCR4 (see previous data, *K*_i_ = 5.2 ± 0.1 × 10^−9^ M), the results suggest that binding of the ligand to the receptor directly induces monomerization. To further assess if the effect of IT1t was consistent with noncovalent binding of the ligand to the receptor and hence was both rapid and reversible, we next treated cells expressing CXCR4–mEGFP with 2 × 10^−8^ M IT1t for 30, 60, or 120 s before cell harvest. Although this did not alter the overall density of molecules of CXCR4–mEGFP in RoIs (Fig. 3*A*), the effect of the antagonist on oligomeric organization was manifest as rapidly as we could arrange to harvest cells after addition of IT1t as assessed by both SpIDA (Fig. 3*A*) and blue native-PAGE (Fig. 3*B*). Notably, the effect of IT1t was also rapidly reversed. Washout of the ligand resulted in restoration of oligomeric organization within 4 min (Fig. 3*C*). Whilst such experiments cannot be analyzed directly to determine association and dissociation binding rates for IT1t from CXCR4–mEGFP because, as shown in Figure 2*B*, IT1t is a high-affinity antagonist, these outcomes emphasize that it must have high rates of both association and dissociation from the receptor.

### The effect of IT1t is selective

This effect of IT1t was selective: A second antagonist of CXCR4 is AMD3100 (Plerixafor), which is used clinically as a treatment for leukemia and solid tumors (24). This ligand has an approximately 10-fold lower affinity for CXCR4 than IT1t (Fig. 3*D*) (IC_50_ of 3.9 × 10^−7^ M for AMD3100 [against an EC_80_ concentration of CXCL12] as compared with 2.6 × 10^−8^ M for IT1t). However, treatment of cells expressing CXCR4–mEGFP with this ligand at concentrations up to 1 × 10^−5^ M had no significant effect on the oligomeric complexity of the receptor measured by either blue native-PAGE (Fig. 3*E*) or by SpIDA (Fig. 3*F* and Table 1).

### FIF spectrometry reinforces conclusions from SpIDA

**Figure 4:**
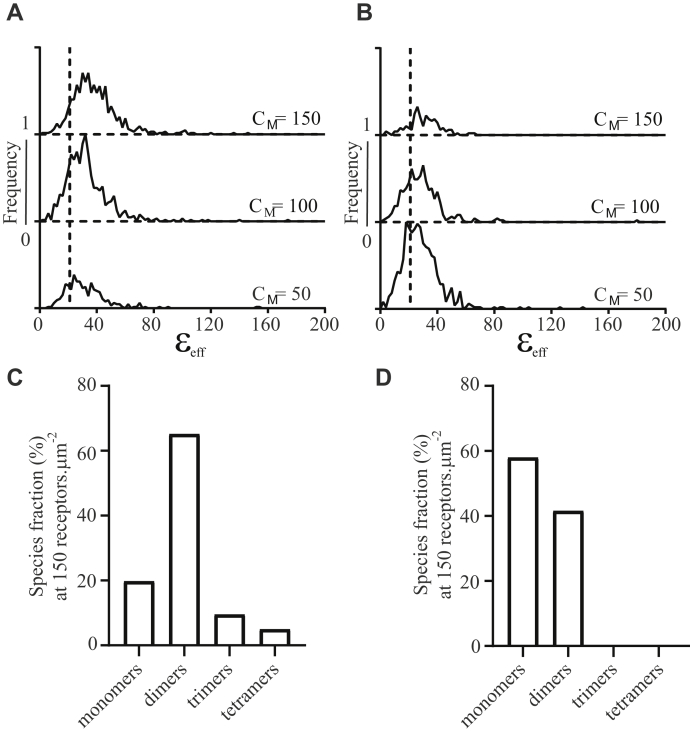
**IT1t treatment disrupts CXCR4 oligomerization: analysis by fluorescence intensity fluctuation spectrometry**. *A and B,* wire graph plots showing how the frequency of occurrence of effective brightness measured from individual segmented RoIs is distributed over a range of different protomer concentration ranges in untreated (*A*) and IT1t-treated cells (2 × 10^−8^ M, for 2 min). Identical concentration ranges were analyzed for each experimental group, and the center of each concentration range analyzed is indicated above the plotted graphs. A vertical dotted line indicates the value for monomer mean brightness used, 20.97. *C,* FIF spectroscopy analysis of cells expressing CXCR4–monomeric enhanced green fluorescent protein at median density of 150 receptors·μm^−2^. *D,* cells treated with 2 × 10^−8^ M IT1t for 2 min, median density 150 receptors·μm^−2^. IT1t, isothiourea-1t.

Although SpIDA has many useful attributes (21), as for related methods including number and brightness analysis (29) and photon counting histogram (30), it only provides information on the average size of oligomers within potentially complex mixtures of oligomers of different sizes and also may be sensitive to brighter inhomogeneities in the fluorescence images. To overcome these issues, we recently developed a new approach described as one- and two-dimensional FIF spectrometry (22, 23). When this approach was applied to images of the basolateral membrane surface of cells induced to express CXCR4–mEGFP at a median density of approximately 150 receptors·μm^−2^, results were consistent with the receptor existing as a mixture of monomers and dimers, with a preponderance of dimers (Fig. 4) and with little evidence of a substantial population of trimers or tetramers (Fig. 4, *A* and *C*). The presence of 2 × 10^−8^ M IT1t for 2 min resulted in FIF spectrometry also revealing a large degree of monomerization of the receptor in this expression range (Fig. 4, *B* and *D*).

### Studies with dimeric interface mutants of CXCR4

**Figure 5:**
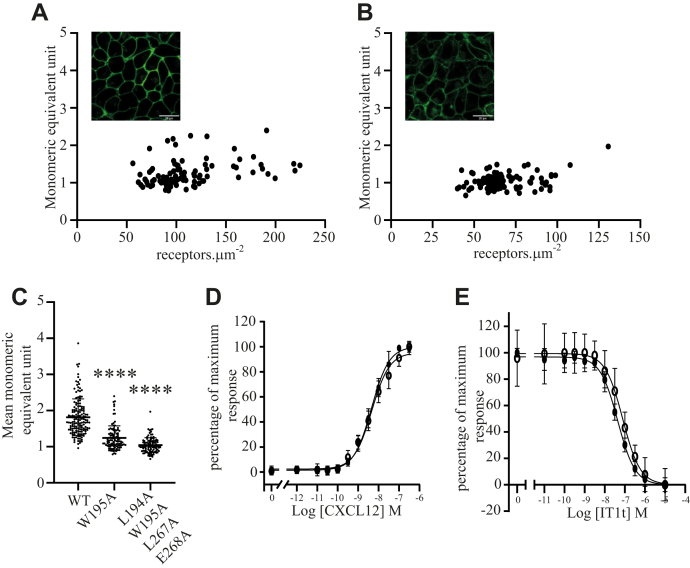
**Analysis of CXCR4 dimer disrupting mutants.***A and B (inset)*, respectively, representative confocal images of cells induced to express Trp^195^AlaCXCR4–monomeric enhanced green fluorescent protein (mEGFP) and Leu^194^Ala, Trp^195^Ala, Leu^267^Ala, Glu^268^AlaCXCR4–mEGFP. The scale bars represent 20 μm. *A,* spatial intensity distribution analysis of cells expressing Trp^195^AlaCXCR4–mEGFP and *B,* Leu^194^Ala, Trp^195^Ala, Leu^267^Ala, Glu^268^AlaCXCR4–mEGFP (see also Table 1). *C,* monomeric equivalent units for wildtype (WT), Trp^195^AlaCXCR4–mEGFP and Leu^194^Ala, Trp^195^Ala, Leu^267^Ala, Glu^268^AlaCXCR4–mEGFP (data are means ± SD [∗∗∗∗different from WT *p* < 0.0001]). *D,* [^35^S]-GTPγS binding assay: the ability of varying concentration of CXCL12 to promote binding of [^35^S]-GTPγS in membranes expressing Trp^195^AlaCXCR4–mEGFP (*closed symbols*) or Leu^194^Ala, Trp^195^Ala, Leu^267^Ala, Glu^268^AlaCXCR4–mEGFP (*open symbols*). *E,* [^35^S]-GTPγS binding assay: the ability of isothiourea-1t to antagonize an EC_80_ concentration of CXCL12 in membranes expressing Trp^195^AlaCXCR4–mEGFP (*closed symbols*) or Leu^194^Ala, Trp^195^Ala, Leu^267^Ala, Glu^268^AlaCXCR4–mEGFP (*open symbols*). *D and E,* combined results of n = 3 experiments.

Certain mutants at the structurally observed dimer interface of CXCR4 including Trp^195^AlaCXCR4 and Leu^194^Ala, Trp^195^Ala, Leu^267^Ala, Glu^268^Ala CXCR4 have been reported to partially interfere with such interactions (7). Initially, we generated each of these variants within the context of CXCR4–mEGFP and again expressed them stably in Flp-In T-REx 293 cells. Following induction, confocal imaging indicated both CXCR4 mutants to be present largely at the plasma membrane (Fig. 5*A* and *B* insets), although the quadruple Leu^194^Ala, Trp^195^Ala, Leu^267^Ala, and Glu^268^AlaCXCR4 mutant appeared to be less well expressed and more intracellular than the single Trp^195^AlaCXCR4 mutant. SpIDA confirmed the lower expression level of the quadruple mutant (Fig. 5, *A*–*B* and Table 1) and, in addition, that the mean level of expression of Trp^195^AlaCXCR4–mEGFP was significantly lower than for wildtype CXCR4–mEGFP (Table 1). In both cases, the assessed oligomeric complexity was also substantially lower than of wildtype CXCR4–mEGFP. In the case of Trp^195^AlaCXCR4–mEGFP, the mean monomeric equivalent unit (MEU) was 1.24 ± 0.34 and for the quadruple mutant 1.00 ± 0.21 (means ± SD; n = 100 RoIs) (Fig. 5*C*) consistent with this mutant being essentially monomeric at these expression levels. Despite this mutant being monomeric, CXCL12 was still effective in promoting binding of [^35^S]GTPγS and with similar potency (Fig. 5*D*) as the wildtype receptor. This indicates that, in cell membranes, a monomeric form of this receptor is adequate to allow activation of G_i_-family G proteins and that signaling does not require receptor dimerization. Notably, IT1t was again effective in blocking the action of CXCL12 (Fig. 5*E*) at both mutants and with affinity (IC_50_ Trp^195^Ala CXCR4–mEGFP = 4.4 × 10^−8^ M, Leu^194^Ala, Trp^195^Ala, Leu^267^Ala, Glu^268^Ala CXCR4–mEGFP = 7.8 × 10^−8^ M) (Fig. 5*E*) similar to the wildtype receptor, indicating negligible co-operative binding of IT1t to the individual protomers in the dimer.

### IT1t promotes dimerization of a largely monomeric nonsignaling mutant of CXCR4

**Figure 6:**
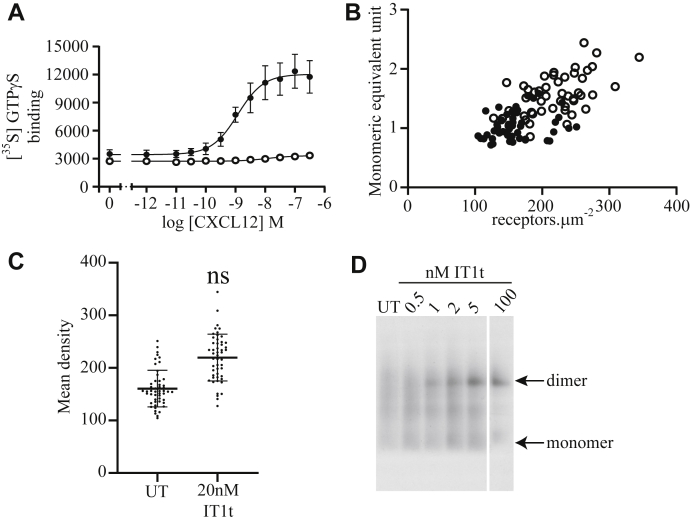
**IT1t increases the oligomeric organization of a signaling defective mutant of CXCR4**. *A,* membranes prepared from cells induced with 100 ng.ml^–1^ doxycycline (24 h) to express CXCR4–monomeric enhanced green fluorescent protein (mEGFP) (*closed circles*) or Asn^119^LysCXCR4–mEGFP (*open circles*) were exposed to the agonist CXCL12 at the concentrations indicated. [^35^S]-GTPγS binding was determined and plotted as dpm [^35^S]-GTPγS (wildtype EC_50_ = 1.19 × 10^–9^ M, Asn^119^LysCXCR4–mEGFP EC_50_ = 1.16 × 10^−8^ M). *B,* spatial intensity distribution analysis of cells expressing Asn^119^LysCXCR4-mEGFP untreated (*open circles*) or treated with 2 × 10^−8^ M IT1t for 1 to 3 h (*closed circles*), for details see Table 1. *C,* receptor density with and without treatment with 2 × 10^−8^ M IT1t (untreated [UT] = 160.6 ± 4.9, 2 × 10^−8^ M IT1t = 219.6 ± 6.3 receptors.μm^−2^, means ± SD; *p* > 0.05; not significantly different [NS]). *D,* blue native-PAGE of lysates of cells expressing Asn^119^LysCXCR4–mEGFP after treatment with varying concentrations of IT1t for 1 h. Monomer and dimer bands as indicated. IT1t, isothiourea-1t.

Certain mutants of CXCR4 have been reported to affect the ability of the receptor to respond to CXCL12. These include mutations of Asn^119^ (positional identification residue 3.35) (31, 32, 33). Whilst, as noted earlier, CXCL12 promoted binding of [^35^S]GTPγS to membranes expressing wildtype CXCR4–mEGFP in a concentration-dependent manner, this was not the case for Asn^119^Lys CXCR4–mEGFP (Fig. 6*A*). SpIDA indicated Asn^119^Lys CXCR4–mEGFP to be largely monomeric in the absence of ligand treatment (Fig. 6*B*), but treatment with IT1t increased (*p* = 0.0001) oligomeric complexity without increasing (*p* > 0.05) levels of the receptor construct (Fig. 6*C*). Blue native-PAGE again indicated the appearance of an apparently dimeric species of Asn^119^Lys CXCR4–mEGFP in the presence of IT1t (Fig. 6*D*).

## Discussion

Previous investigation of the oligomeric structure of the human CXCR4 receptor has suggested that it exists, at least in part, as a dimer (7,15, 16, 17, 18). Moreover, atomic-level X-ray structures have also shown organization in which the receptor appears to dimerize (7). The latter does not necessarily indicate that receptors in the cell membrane form dimers as conditions in a crystal and in a membrane are very different but are clearly consistent with this possibility. To investigate the oligomeric structure of the CXCR4 receptor, initially we used SpIDA (24, 25, 34, 35). This was supplemented by using a recently developed FIF spectroscopy technique (22, 23) that is also based on analysis of fluorescence fluctuations. The results of these analyses showed a strong propensity for the CXCR4 receptor to form dimers and even higher-order oligomers that were related to the level of expression and receptor density. Oligomerization became more complex with increased expression, suggesting an effect of mass action. However, significant levels of higher-order organization above dimers were largely restricted to receptor levels beyond those found in either normal or pathophysiological conditions and at receptor expression level higher than studied by others (19, 36). To reinforce such conclusions, we employed blue native-PAGE as a completely different approach (37). This method involves lysing cells under conditions that can preserve protein oligomeric structure (similar to the detergent *n*-dodecyl β-*d*-maltoside) rather than destroying it as often occurs with SDS–PAGE gels. Gels are run with no SDS or reducing agent, and instead proteins are given a charge by binding G250, similar to Coomassie brilliant blue. Wildtype CXCR4 was found to migrate as two separate bands, and whilst it is difficult to ascribe molecular mass with confidence with this technique, the results, particularly when comparing untreated samples with those exposed to IT1t, provided confidence to assign the larger band to dimers/oligomers and the lower band to monomers.

An important question in these studies was whether ligand binding altered oligomeric organization and, as a corollary, whether the oligomerization state would affect ligand binding or function. To explore this question, we used two specific CXCR4 antagonists that have distinct chemical structures, IT1t and AMD3100. Only IT1t was found to influence organization of the wildtype receptor and caused almost complete monomerization at concentrations consistent with receptor binding, and this was manifest rapidly and was reversed when the ligand was removed. By contrast, AMD3100 had no significant effects, even at concentrations markedly higher than predicted to result in virtually full receptor occupancy. Blue native-PAGE analysis was again consistent with the data based on FIF.

Other studies have explored whether the binding of various CXCR4 blockers might alter receptor dimerization. In particular, Lao *et al*. (19) reported that exposure to AMD3100 could increase the dimer fraction. However, as these studies employed single-molecule imaging, they were restricted to analysis of very low receptor expression levels at which the receptor was largely monomeric in the untreated state. Moreover, although high concentrations of AMD3100 produced a trend toward dimerization, it was unclear if this was indeed statistically significant. More clearly, whilst the current article was under review, Işbilir *et al*. (36) reported, as we do, that AMD3100 is unable to alter the dimeric proportion of CXCR4. Equally, as we do, they noted that IT1t was highly effective at causing monomerization of CXCR4 and did so rapidly (36). We have championed the use of SpIDA to monitor GPCR quaternary organization for a number of years (21, 25, 26, 34, 35), and it is therefore gratifying to note that Işbilir *et al*. (36) have now also adopted this approach and in so doing have generated results strongly in concordance with our own observations.

It is unclear why different blockers of CXCR4 have such distinct effects on the ability of protomers of the receptor to interact. Although details of how IT1t interacts with the receptor are defined in the available atomic-level structures (7), such static snapshots are unable to provide insights into potential linked conformational changes. Moreover, although Işbilir *et al*. (36) modeled possible modes of binding of a range of CXCR4 blockers that either promoted monomerization or did not, these also do not provide direct insight into mechanism. It is possible that, in time, use of molecular dynamics simulations could provide a useful perspective.

Various mutations introduced into CXCR4 have been reported to affect either its signaling (31, 32, 33), dimerization potential (38), or to alter its ability to promote activation of G_i_-family G proteins (33). We thus assessed a number of such mutants for their ability to form oligomers and examined the effects of both CXCL12 and of IT1t upon them to assess any potential role of dimers of CXCR4 in signaling and function. These mutants included alterations toward the top of the fifth transmembrane domain, Trp^195^Ala and at the top of the fifth and sixth transmembrane domains, Leu^194^Ala, Trp^195^Ala, Leu^267^Ala, Glu^268^Ala, based on a proposed dimerization interface (38). Whilst both these mutants were present at the cell surface, their expression levels were reduced in comparison to wildtype, the quadruple mutant was almost completely monomeric. Nevertheless, this mutant was able to activate G_i_-proteins in response to CXCL12 with similar potency as wildtype and to bind IT1t with similar affinity. The strictly monomer state of the Leu^194^Ala, Trp^195^Ala, Leu^267^Ala, Glu^268^AlaCXCR4 mutant, whereas at significant expression levels, the wildtype CXCR4 receptor is essentially dimeric also therefore allows us to conclude that both monomer and dimer forms of CXCR4 are able to bind the chemokine CXCL12 and generate G protein–mediated signals with similar potency. It is of course well established that defined monomers of other rhodopsin-like GPCRs are able to bind and activate G proteins in an agonist-dependent manner (39). However, this key question is one in which our results and conclusions differ from those of Işbilir *et al*. (36), as they suggested that, at least in terms of basal activity, dimerization might play an important role. We did not directly explore the contribution to basal activity of CXCR4 dimerization, in part because the monomeric Leu^194^Ala, Trp^195^Ala, Leu^267^Ala, Glu^268^AlaCXCR4 mutant was relatively poorly expressed, whilst Işbilir *et al*. (36), in contrast did not actively explore effects of monomeric *versus* dimeric status on agonist-mediated functions and thus the data sets are not directly able to compare these functional characteristics.

We also explored the mutant Asn^119^Lys CXCR4. Located within the third transmembrane domain, Asn^119^Lys CXCR4 responded poorly to CXCL12 but still bound IT1t with high affinity. Notably, although largely monomeric in the basal state, addition of IT1t was here observed to promote receptor dimerization. Thus, although the Asn^119^Lys alteration appears to limit oligomerization, this can then be overcome by the binding of IT1t, which in this example appears to stabilize the dimeric state.

Overall, these studies indicate the plasticity of CXCR4 organization that alters with receptor density and upon binding certain but not all antagonist ligands. Moreover, both mutations that were designed to interfere with receptor dimerization and other mutants where this may not have been the anticipated outcome can clearly affect the propensity of the CXCR4 receptor to form dimers and oligomers.

## Experimental procedures

### Materials

General laboratory chemicals were from Sigma–Aldrich or Fisher Scientific. Otherwise, DNA restriction endonucleases, calf intestinal alkaline phosphatase, T4 DNA polymerase, and T4 ligase were from New England Biolabs. NuPage Novex precast 4% to 12% Bis–Tris gels, NuPage Mops SDS running buffer, NativePAGE Novex 3% to 12% Bis–Tris gels and associated reagents were from Invitrogen. QIAfilter Plasmid Maxi Kit, plasmid miniprep kit, PCR purification kit, and QIAquick gel extraction kit were from Qiagen. Agarose was from Bio-Rad. Secondary horseradish peroxidase–conjugated antibody was from Sigma–Aldrich or GE Healthcare Life Sciences. Enhanced chemiluminescence reagent was purchased from Pierce. Polyethylenimine was from Polysciences, Inc. LI-COR reagents, phosphate-buffered saline (PBS) blocking buffer, and fluorescent-conjugated secondary antibodies were from LI-COR Biosciences. Protease inhibitor cocktail tablets were from Roche Diagnostics. Antibodies were either generated in house (anti-GFP) or were from Abcam (antitubulin). Recombinant human CXCL12 (SDF1α) was from Pepro Tech. IT1t dihydrochloride and AMD3100 octahydrochloride were from Tocris.

### Plasmid constructs

Human CXCR4 was fused in frame at its C-terminal position to monomeric (m, Ala^206^Lys) EGFP by subcloning after PCR amplification (using primers designed to add Xho1 and EcoR1 sites) into the Xho1 and EcoR1 sites of pEGFP-N1, which had been previously modified to include the monomeric Ala^206^Lys mutation. This yielded the plasmid CXCR4–mEGFP. The Asn^119^Lys, Trp^195^Ala and Leu^194^Ala, Trp^195^Ala, Leu^267^Ala, Glu^268^Ala mutations were introduced into CXCR4–mEGFP using the Dpn1/Quickchange approach as described (40). To make Flp-In T-REx cell lines, the CXCR4–mEGFP inserts were subcloned into the EcoRV and Not1 sites of pcDNA5–FRT–TO by cutting with Nhe1, blunting the sticky end with T4 DNA polymerase, and then cutting with Not1. The fragments were then ligated into pcDNA5–FRT–TO. All constructs were verified by sequencing prior to use.

### Cell lines

All cells were maintained in a humidified incubator with 95% air and 5% CO_2_ at 37 °C. Parental Flp-In T-REx 293 cells (Invitrogen) were maintained in Dulbecco's modified Eagle's medium (high glucose) supplemented with 10% (v/v) fetal bovine serum, 100 U ml^−1^ penicillin, 100 μg ml^−1^ streptomycin, 10 μg ml^−1^ blasticidin, and 100 μg ml^−1^ zeocin. Cell lines generated that used Flp-In T-REx 293 cells as the base were maintained in Dulbecco's modified Eagle's medium (high glucose) supplemented with 10% (v/v) fetal bovine serum, 100 U ml^−1^ penicillin, 0.1 mg ml^−1^ streptomycin, 10 μg ml^−1^ blasticidin, and 200 μg ml^−1^ hygromycin B.

### Stable cell line generation

Inducible Flp-In T-REx 293 stable cell lines able to express PM-1–mEGFP and CXCR4–mEGFP and its mutant derivatives were generated as follows; basal Flp-In T-REx 293 cells were grown to approximately 60% confluency and cotransfected with the desired plasmid and pOG44 at a ratio of 7.2 μg pOG44 to 0.8 μg of the pcDNA5–FRT–TO derivative. Transfections were done using polyethylenimine (41). Cells were propagated in medium containing no selective antibiotic for 2 days. After 48 h, the medium was changed to that without zeocin but supplemented with 200 μg ml^−1^ hygromycin to initiate selection of stably transfected cells. Pools of cells were established (10–14 days for resistant colonies to form) and tested for inducible expression by the addition of 100 ng.ml^−1^ doxycycline for 48 h followed by screening for fluorescence corresponding to mEGFP and by immunoblotting.

### Generation of cell lysates and immunoblotting

Cells were washed once in cold PBS (120 mM NaCl, 25 mM KCl, 10 mM Na_2_HPO_4_, and 3 mM KH_2_PO_4_; pH 7.4) and harvested with a minimum volume ice-cold lysis buffer (1 × radioimmunoprecipitation assay buffer), (50 mM Hepes,150 mM NaCl, 1% Triton X-100, 0.5% sodium deoxycholate, 10 mM NaF, 5 mM EDTA, 10 mM NaH_2_PO_4_, 5% ethylene glycol; pH 7.4) supplemented with complete protease inhibitor mixture (Roche Diagnostics). Extracts were passed through a 25-gauge needle and incubated for 30 min at 4 °C while on a rotating wheel, centrifuged for 10 min at 21,000×*g*, and the supernatant recovered to fresh tubes. Samples were prepared by the addition of SDS–PAGE sample buffer and heated to 65 °C for 5 min before being subjected to SDS–PAGE analysis using NuPAGE 4% to 12% Bis–Tris gels and Mops buffer. After separation, the proteins were electrophoretically transferred to nitrocellulose membrane, which was then blocked (5% fat-free milk powder in PBS with 0.1% Tween-20) at 4 ºC on a rotating shaker overnight. The membrane was incubated for 3 h with primary antibody (1:10,000 sheep anti-GFP, with or without as indicated 1:10,000 mouse antitubulin) in 2% fat-free milk powder in PBS–Tween, washed (3 × 10 min PBS–Tween) and then incubated for 3 h with appropriate secondary antibody (horseradish peroxidase–linked rabbit antigoat IgG, diluted 1:10,000 in 2% fat-free milk powder in PBS–Tween with or without horseradish peroxidase–linked sheep antimouse secondary at 1:10,000). After washing as mentioned previously, signal was detected by enhanced chemiluminescence (Pierce Chemical) according to the manufacturer's instructions. Alternatively, some blots were detected using fluorescently labeled secondary antibodies (IRDye 800CW donkey antigoat and IRDye 680RD donkey antimouse [Li-Cor Biotechnology]). These were used at a dilution of 1:20,000 in Odyssey PBS blocking buffer (Li-Cor Biotechnology) according to the manufacturer's instructions. Secondary antibodies were detected using an Odyssey Sa Infra-red Imaging System (Li-Cor Biotechnology).

### Blue native-PAGE

Flp-In T-REx 293 cells induced with doxycycline to express constructs as indicated, subject to treatment as indicated, were harvested in 1× PBS and lysed in lysis buffer (150 mM NaCl, 0.01 mM Na_3_PO_4_, 2 mM EDTA, 0.5% *n*-dodecyl β-*d*-maltoside, and 5% glycerol supplemented with protease inhibitor cocktail tablets, pH 7.4) on a rotating wheel for 30 min at 4 °C. Samples were then centrifuged for 30 min at 100,000×*g* at 4 °C, and the supernatants collected. About 16 μg of solubilized supernatant plus 5 μl G250 additive was loaded on to each lane of NativePAGE Novex 3% to 12% Bis–Tris gels. After electrophoresis at 0 °C (using buffers and conditions indicated by the manufacturer), proteins were transferred (90 min at 25 V) on to a polyvinylidene fluoride membrane, which had been prewetted for 30 s in methanol and then soaked for several minutes in transfer buffer. The membrane was then fixed in 8% acetic acid, shaking for 15 min, stained with Ponceau S (Sigma–Aldrich) (0.2% in 1% acetic acid) to allow the markers to be visualized, rinsed to remove the Ponceau S, and immunoblotted with anti-GFP antiserum as described previously.

### Membrane preparation

Membranes were generated from Flp-In T-REx 293 cells treated with 100 ng ml^−1^ doxycycline to induce expression of the construct of interest. Cells were washed with ice-cold PBS, removed from dishes by scraping, and centrifuged at 3000 rpm for 5 min at 4 °C. Pellets were resuspended in TE buffer (10 mM Tris–HCl, 0.1 mM EDTA; pH 7.5) containing a protease inhibitor mixture (Roche Applied Science) and passed through a 25-gauge needle 10 times before being homogenized with a 5 ml Teflon-on-glass hand-held homogenizer. The lysate was then passed through the needle for a further 10 times. This material was centrifuged at 1200 rpm for 5 min at 4 °C, and the supernatant was further centrifuged at 50,000 rpm for 30 min at 4 °C. The resulting pellet was resuspended in TE buffer, and protein content was assessed using a BCA protein assay kit (Pierce, Fisher Scientific).

### [^35^S]GTPγS binding assay

About 10 μg of membrane protein was preincubated for 15 min at 30 °C in assay buffer (20 mM Hepes; 5 mM MgCl_2_; 160 mM NaCl; 0.05% bovine serum albumin; pH 7.5) containing the indicated ligand concentrations. The reaction was then initiated with addition of assay mix resulting in the following final concentrations: [^35^S]GTPγS (50 nCi per tube); 1 μM GDP, and 30 μg.ml^−1^ saponin. The reaction was terminated after 45 min of incubation at 30 °C by rapid filtration through UniFilter GF/C filter plates using a 96-well Filtermate cell harvester (PerkinElmer Life Sciences). Unbound radioligand was removed from filters by three washes with ice-cold PBS (pH 7.4) and filters were dried for 2 to 3 h at room temperature. About 50 μl of Microscint-20 (PerkinElmer Life Sciences) was added to each well of the dried filter plates, which were then sealed, and [^35^S]GTPγS binding was determined by liquid scintillation spectrometry. Results were analyzed using GraphPad Prism 8 (GraphPad Software Inc), and this was used to determine EC_50_, EC_80_, IC_50_, and *K*_i_ values.

### SpIDA and FIF spectrometry

#### Cell sample preparation

Inducible Flp-In T-REx 293 stable cell lines able to express PM-1–mEGFP, CXCR4–mEGFP, and its mutant forms were seeded on to poly-*D*-lysine–coated circular glass coverslips (size = 30 mm) at a density of 2.5 × 10^5^ cells/coverslip. After 24 h of growth, doxycycline (100 ng ml^−1^) was added to the tissue medium to induce construct expression. The cells were grown overnight and were then rinsed and resuspended in Hepes-buffered saline solution (130 mM NaCl, 5 mM KCl, 1 mM CaCl_2_, 1 mM MgCl_2_, 20 mM Hepes, and 10 mM D-glucose, pH 7.4) prior to vehicle or test ligand addition. Coverslips were loaded into an imaging chamber and placed on the microscope stage for image acquisition. When short-term ligand treatment was required, cells were grown in four-well chamber slides (Lab-Tek 4 chamber coverglass; Nunc), treated as required and then fixed in 4% paraformaldehyde for 20 min at room temperature. The cells were then washed 3 times in 1 × PBS and stored under 1 × PBS at 4 °C until required.

#### Confocal image acquisition

Single images in 1024 × 1024 pixels format were recorded upon excitation using the 488-nm laser line of the Zeiss 880 laser scanning confocal microscope (inverted configuration). A 63× plan apochromat oil immersion lens with a numerical aperture of 1.4 was used to record high-resolution images with a lateral pixel size of 0.09 μm and a pixel dwell time of 16.48 μs/pixel. Emitted fluorescent light (505-nm long pass filter) was detected using a photomultiplier tube using the following parameter settings: gain = 850 V, offset = 0, and amplifier gain = 1. The pinhole was set to 1.00 Airy unit, and the laser intensity power was always set to 0.4% to minimize photobleaching and ensure consistency. The 488-nm laser beam waist radius size, photomultiplier tube shot noise, and white noise background signal were quantified as previously detailed (25, 26).

### SpIDA

The MATLAB Graphical User Interface program for implementing SpIDA was downloaded from the neurophotonics software Web site (https://neurophotonics.ca/software), and SpIDA was performed as previously published (35). All RoI measurements were selected from the basolateral membrane surface. MEU values for CXCR4–mEGFP and mutants were measured by normalizing their assessed QB values with an average QB value measured from the PM-1–mEGFP construct. To demonstrate that PM-1–mEGFP was expressed only as monomeric and not as dimeric/oligomeric species, PM-1–mEGFP MEU occurrence/frequency *x* to *y* graphs (MEU bin size = 0.2) were plotted for each MEU value measured during excitation. Such plots revealed a symmetrical distribution of the values, and GraphPad Prism normality tests indicated the distributions were Gaussian (see Results and statistical analyses). The data from each frequency of *x* to *y* plot of CXCR4–mEGFP and mutants were then divided at an MEU value of 1.324 (which represented 86.6% of the data set, falling within the mean +1.5 standard deviations [SDs]), which was set as the border to distinguish between monomeric and dimeric species in studies where individual MEU values exceeded 1.324.

#### Calculation of receptor protomer concentration at the cell surface by SpIDA

The SpIDA software program reports the mean fluorescence intensity for each RoI analyzed. To determine the average receptor concentration within each RoI, an apparent number of particles in the beam area was first calculated by dividing the mean fluorescence intensity for an ROI by the monomeric QB, as follows:

(1) $$N_{SpIDA}\;=\;\frac{\left[\text{I}\right]}{QB_{of\;PM-1x-mEGFP}}$$

Here [I] represents the experimental CXCR4 RoI mean fluorescence intensity value and $QB_{of\;PM-1x-mEGFP}$ represents the measured monomeric QB value obtained from the monomeric molecular brightness reference calibration PM-1–mEGFP sample. The value derived from Equation 1 was then used to determine the total protomer concentration (*i.e.*, number of molecules.μm^−^^2^) of CXCR4–mEGFP and mutant molecules, as seen in Equation 2 below:

(2) $$C_{correctSpIDA}\;=\;\frac{N_{SpIDA}\cdot \gamma }{\int \int \text{PSF}\left(x,y\right)dxdy}$$

Here γ represents a coefficient that depends on the shape of the laser point spread function (PSF) (approximated using a Gaussian–Lorentzian profile) as well as the geometry of the sample (42, 43). For measurements on the basal lateral membrane of cells using a laser beam with an assumed Gaussian–Lorentzian profile, the gamma factor is approximated to be *γ* = 0.5. In Equation 2, ∬PSF(x,y)dxdy is an area-like quantity that represents the size of the physical region from which a fluorescence signal of [I] would be generated if all particles in the beam generated signal as if they were located at the center of the beam. It should be noted that the actual size of the excitation region that contains molecules that make contributions to the measured fluorescence is larger than the area calculated by the integral (44) because the signal level of the fluorescent molecules drops off as they move away from the center of the beam. The area term, ∬PSF(x,y)dxdy, and γ factor are needed to compensate for the nonuniformity of the fluorescence signal from molecules located at various positions within the laser beam and the effective steepness of the boundary defining the excitation volume. Assuming a Gaussian–Lorentzian shaped beam, the area integral over the PSF can be solved analytically:

(3) $$\iint \text{PSF}\left(x,y\right)dxdy\;=\;\frac{1}{2}\pi {\left(w_{xy}\right)}^{2}\;=\;0.111\text{ μ}{\text{m}}^{2}$$

where a value of $w_{xy}=0.2656\;$ was used for the laser beam waist. See Ref. (23).

### FIF spectrometry analysis

The average brightness (and its derived degree of protein oligomerization) reported by SpIDA from entire population of monomers and oligomers with different concentrations and sizes within an RoI is not ideal as an average may not give a true indication of the actual oligomeric content. In contrast to SpIDA, FIF can perform meta-analysis of brightness spectrograms over different protomer concentration ranges to extract the oligomeric species fraction within the sample (*i.e.*, FIF generates oligomer species fraction plots as a function of protomer concentration). FIF analysis was performed using an updated version of a freely available program (https://figshare.com/s/acfd94b21b1105317f56) which consists of three modular steps: (1) selection of RoI and generation of smaller segments; (2) brightness and concentration calculation for each individual segment; and (3) meta-analysis of brightness spectrogram distributions as a function of different concentration ranges. Each module is launched by a separate icon in the graphical user interface toolbar, and in this study, FIF analysis was performed as described (22, 23). A brief description of the use of each module follows. RoI selection and generation of smaller segments (module 1): the individual image files from each experimental group were combined and saved as an image stack prior to importation into module 1 of the FIF spectrometry suite software. The freehand polygon tool was then used to draw multiple RoIs on to the basolateral membrane of each image file. The RoIs from each experimental group were saved and then automatically segmented using a simple linear iterative clustering algorithm to generate smaller square segments (*x*, *y* pixel size = 20 × 20) for spectrometric brightness analysis in modules 2 and 3. Brightness and concentration extraction (module 2): all experimental image data sets were analyzed using Metamorph imaging software to quantify the level of background signal to be subtracted in module 2. Fluorescence intensity values from within each pixel of a defined RoI segment were plotted as a histogram frequency distribution and fit with a single Gaussian model function to obtain a statistical mean and SD value from the pixel fluorescence intensity distribution. The mean and SD values measured from the Gaussian fit along with the signal variance because of the detector (22, 23) were then used to quantify the effective brightness (ε_eff_) and concentration for each analyzed segment. Once the brightness and concentration values have been determined for each segment, brightness frequency distributions as a function of concentration can be visualized either as a (volcano) three-dimensional surface plot of concentration *versus* ε_eff_ or as a wire histogram plot of brightness values derived from different segment bin concentration ranges. Meta-analysis of brightness spectrogram distributions as a function of different concentration ranges (module 3): in the third module, experimental receptor protein brightness distribution values over different concentration ranges were fit with a sum of multiple Gaussian functions. The mean brightness values of each Gaussian peak used in the fitting are linearly related and were set as multiples of the monomeric mean brightness value measured from the monomeric PM-1–mEGFP calibration reference sample. Multiple Gaussian peak fitting of experimental receptor protein brightness spectrogram data sets over a range of different protomer concentrations enabled us to plot oligomer species fraction values as a function of protomer concentration. These plots allowed easy visualization of the different oligomeric receptor protein populations that existed in the analyzed receptor protein experimental samples (data are displayed for a single concentration range).

### Quantification and statistical analysis

Variation in receptor number or mean/median of QB produced by treatment with either ligands or with varying concentrations of doxycycline was assessed by one-way ANOVA, with the use of Bonferroni's or Dunnett's test for multiple comparisons as appropriate. Normality distributions of recovered QB values defined as MEUs were assessed by D'Agostino and Pearson normality tests (at *p* > 0.05) and skewness and kurtosis assessments. Distributions that failed the normality assessment (at *p* < 0.05) were considered to be non-Gaussian.

## Data availability

Images used in this analysis will be deposited at Figshare.

## Conflict of interest

The authors declare that they have no conflicts of interest with the contents of this article.
